# Algorithm based online speech evaluation for Autism - A new horizon

**DOI:** 10.1192/j.eurpsy.2026.10912

**Published:** 2026-07-31

**Authors:** E. Vashdi, R. Popa

**Affiliations:** 1 Yaelcenter, Haifa, Israel; 2 Dinamic Equilibrium, Bucharest, Romania

## Abstract

**Introduction:**

One of the most common characters of the ASD phenomenon is the speech difficulties. The diagnostic tools don’t usually explore the origin of the speech problem but rather describe it superficially. Research show that the prevalence of speech problem among ASD is very high. One of the researches found that 63% of the children diagnosed with ASD has Childhood apraxia of speech (CAS). ASD prevalence today is 1:54 in the USA (2020). Based on that, we can conclude that at least 1 percent of the population have severe speech problems.

**Objectives:**

Since the prevalence of speech motor problems is so high and the importance of speech as a functional tool is high as well, there is a need for a treatment tool which will be able to address the growing need. The VML method was developed and designed to treat CAS focusing on the ASD population. After experiencing over 3000 children in many countries around the world, we have developed an algorithm which represents the VML analysis process. The algorithm includes almost 1000 /If…Then../ conditions and was found reliable with copying the in-person VML evaluation. The algorithm generates a treatment program with 95% accuracy of the elected treatment topics.

**Methods:**

Based on the algorithm, we have developed a software which can produce a highly detailed motor speech treatment program. The software is web based, available now in English and soon in other languages as well. The user is required to fill in the speech data using the software interface.

**Results:**

The uniqe software gives families and therapists all over the world the opportunity to use the VML method remotely in minimal cost. The program includes main treatment topics, detailed exercises, picture and videos demonstrating the proposed treatment and general guidelines. The software users are supported by the VML experts around the world.

**Conclusions:**

The MYVML evaluation software is innovation in the field of speech treatment, striving to share the knowledge and give the treatment tool to as many practitioners and families as possible.

This lecture will present the VML method background, software structure, analysis and functionality through demonstrations of the software.

**Disclosure of Interest:**

None Declared

